# Red blood cell folate as a risk factor for breast cancer among patients at a tertiary hospital in Uganda: a case control study

**DOI:** 10.1186/1477-7819-12-260

**Published:** 2014-08-18

**Authors:** Gideon Rukundo, Moses Galukande, Peter Ongom, Jane Odubu Fualal

**Affiliations:** Surgery department, Mulago National Referral Hospital, Mulago Hill Road, P. O. Box 7072, Kampala, Uganda; Surgery department, College of Health Sciences, Makerere University, Mulago Hill Road, P. O. Box 7051, Kampala, Uganda

**Keywords:** Red blood cell, Folate, Breast cancer

## Abstract

**Background:**

Folate has been shown to play a complex but unclear role in carcinogenesis, with some studies showing that low folate intake protects against early carcinogenesis while high folate intake promotes advanced carcinogenesis. Other studies have shown that high folate is associated with decreased breast cancer risk and overall survival, yet others found no such association.

This study therefore sought to determine the association between red blood folate levels and breast cancer among women seen at a tertiary Ugandan hospital.

**Methods:**

A case control study was conducted where female patients with a histological diagnosis of breast cancer were recruited as cases, and females without cancer attending other surgical clinics as controls. Demographics and social behavior data were collected and 5mls of blood drawn for laboratory testing of red blood cell (RBC) folate, serum vitamin B12 and RBC count. Ethical approval was obtained.

**Results:**

In this study, a total of 145 women were recruited as 72 cases and 73 controls. The odds of having breast cancer among women with normal folate levels compared to those with low folate levels were 1.4 (95% CI 0.7 to 2.9) *P* = 0.290. Ninety participants (63%) had low RBC folate and 53 participants (37%) had normal RBC folate. Thirty five (45%) of the women from a rural setting had normal folate levels compared to 18(28%) women from an urban setting.

**Conclusions:**

There was no significant association found between RBC folate and breast cancer among this group of women in Uganda.

## Background

Folate, a B vitamin (B_9_) found naturally in many food sources, particularly in dark green leafy vegetables [1], is essential for regenerating methionine, the methyl donor for DNA methylation, and for producing the purines and pyrimidine thymidylate required for DNA synthesis and repair [2]. The role of folate is well documented in the prevention of neural tube defects and is increasingly studied in relation to cardiovascular disease risk [3–5]. Evidence for its potential role in carcinogenesis is encouraging, although the effect is less well studied [3].

Numerous studies have examined the association between folate and both colorectal and lung cancers, but few have been conducted for breast cancer, even in the western world [3], and there is as of yet, no consensus on the association.

In this study, we investigated the association between low red blood cell (RBC) folate levels and breast cancer. Most studies have looked at high dietary folate food as a protective factor against breast cancer and most of these studies were carried out in countries where either folate fortification is performed or the incomes and nutritional status of participants are higher than in Uganda.

## Methods

### Study setting and design

This was a hospital-based case control study. The study was carried out at the Breast Clinic at Mulago National Referral and Teaching Hospital of Makerere, University College of Health Sciences, Uganda.

Mulago Hospital is located in Kampala, the capital city of Uganda. Patients seen in Mulago Hospital are referred from all over the country; however a significant number of patients are self-referred.

### The cases

The study included patients attending the Breast Clinic with histologically confirmed breast cancer and not receiving chemotherapy.

Patients who were 18 years of age and above were included because breast cancer is rare below this age. Secondly, 18 years is the minimum age in Uganda at which written and informed consent is obtained.

Patients who were pregnant, taking folic acid or multivitamin supplementations containing folic acid, taking anticonvulsants, and patients with a history of menorrhagia (menstruation lasting more than seven days) were excluded.

### The control patients

The control patients were without breast cancer or any other cancer (verified history and clinical examination). They were recruited from surgical outpatient clinics other than the Breast Clinic. Excluded from the control group were pregnant women, participants taking folic acid supplementation or multivitamins containing folic acid, patients taking anticonvulsants, or those with a history of menorrhagia (menstruation lasting more than 7 days. Also excluded were those with benign breast conditions.

### Study variables

The following were considered: RBC folate level, age, parity, age at menarche and menopause, Body Mass Index (BMI), residence (rural versus urban), use of hormonal contraception, tobacco use versus no tobacco usage, alcohol consumption, hemoglobin level, and serum vitamin B12 level.

### Study procedure

The purpose of the study, the assessment, and sample collection procedures were carefully explained to each patient individually. Participants were allowed to ask questions freely to ensure that they understood the intention of the study.

Screening for suitability included history taking and physical examination.

Written informed consent was obtained from those who satisfied the inclusion criteria and they were subsequently enrolled in the study on a consecutive basis. Those who did not meet the inclusion criteria as well as those who declined to participate in this study were offered the care that had been scheduled.

Under aseptic conditions, 5mls of blood from each participant was drawn from the antecubital vein. Following this, 3mls were put into a red top vacutainer and 2mls were put into a purple top vacutainer containing ethylenediaminetetraacetic acid (EDTA). The samples were transported to the lab for processing at below room temperature and within two hours. Blood samples were stored in a freezer at -20°C to ensure that they were available in case the need for re-examination arose in the near future.

Samples were processed by using the COBAS e601 analyzer (Roche diagnostics, Indianapolis, USA) and two lab technologists processed the samples.

### Data collection and analysis

Interviews were conducted using a pretested questionnaire and laboratory results were recorded. The laboratory result forms were safely and confidentially stored.

Data was double entered using Epidata version 3.1 (Epidata Association, Odense, Denmark). The data was checked for range and consistency. Data was transferred to STATA version 11.0 (StataCorp LP, Texas, USA for analysis and all *P* values were two tailed and *P* values of <0.05 were considered to indicate statistical significance. Categorical data was displayed as frequencies and percentages.

The primary exposure dependent variable was RBC folate level which was categorized into low folate (470.9 ng/ml or less) and normal folate (471to 1289 ng/ml). The outcome variable was breast cancer which was treated as a binary variable: breast cancer cases and controls. All continuous variables were arranged into categories to allow usage of the Chi squared test.

Age was grouped into five categories: 18 to 39, 40 to 49, 50 to 59, 60 to 69, and 70 and above. Social and cultural behavioral factors which were assumed to influence participant’s dietary patterns were mainly determined by looking at residence, which were categorized as rural versus urban. Body Mass Index was categorized as underweight (less than 18.5 kg/m^2^), normal (18.5 to 24.9 kg/m^2^), overweight (25 to 29.9 kg/m^2^), and obese (30 kg/m plus^2^). Parity was categorized as nulliparous (zero), low parity (one to four), and mutiparity (five and above). Menopausal status was binary; pre-menopausal and post-menopausal. Menopause was defined as age at which the participant ceased menstruation plus one year.

Hemoglobin level was categorized according to the World Health Organization (WHO) guidelines for non-pregnant women over the age of 15 years [6]; severe anaemia (<8 g/dl), moderate anaemia (8 to 10.9 g/dl) mild anemia (11 to 11.9 g/dl) and normal (12 g/dl and above).

The other variables were categorized according to whether the participant was identified with the variable or not. Chi-square and logistic regression were used to examine the association between RBC folate levels and breast cancer. Bivalent association between other factors and having breast cancer was examined and presented as odds ratio, and multivariate logistic regression was used to look for potential confounders (age, parity, residence, B_12_, BMI, alcohol, tobacco use and contraceptive use).

### Quality control

Pre- testing of the questionnaire was done before the study was carried out.

All patients were fully screened for inclusion and exclusion according to the criteria that was set.

One laboratory was used and all tests were done by two laboratory technologists experienced in using the COBAS e601, for RBC folate and vitamin B12 tests.

Calibration of the COBAS e601 was done before samples (which had been stored as outlined above) were processed.

Samples were kept in a freezer (Defy Kirsch, Germany) at -20°C in the clinical chemistry laboratory for future re-evaluation if need arose.

### Ethical consideration

Institutional ethical approval was obtained from the Ethics and Research Committees of Makerere University College of Health Sciences. Written informed consent was obtained from each study participant.

## Results and discussion

Seventy two cases of breast cancer and 73 controls were enrolled in the study from January 2012 to May 2012. Two cases were not included in the analysis due to missing laboratory results. Study participants were compared for study variables and results are shown in Table 1.

**Table 1:** **Comparing variables of breast cancer cases and the controls**

Characteristics n = 145	Cases		Controls		***P***values*
	n = 72	%	n = 73	%	
**Age**					
20 – 39	15	21	15	21	
40 – 49	13	18	15	21	
50 - 59	23	23	23	23	
60 - 69	11	15	11	15	
70+	10	14	9	12	0.996
**Residence†**	70		73		
Rural	41	59	38	52	
Urban	29	41	35	49	0.433
**Body Mass Index**					
Underweight	1	1	4	6	
Normal	38	53	29	40	
Overweight	18	25	25	34	
Obese	15	21	15	21	0.247
**Contraceptive use**					
Yes	26	37	26	36	
None	45	63	47	64	0.900
**Menopause**					
Pre-menopause	28	39	31	43	
Post-menopause	44	61	42	57.5	0.661
**Parity**					
Null	6	8	1	1	
Low parity	23	32	19	26	
Multi parity	43	60	53	73	0.083
**Alcohol use**					
Yes	43	60	30	41	
No	29	40	43	59	0.25
**Taking tobacco**					
Yes	5	7	9	13	
No	67	93	63	88	0.261

*Chi square = *P* values.

Percentages may be = 100 due to rounding.

†Missing =2.

### Age

The mean age for cases was 51 years (range 23 to 87) and the mean age for controls was 52 years (range 23 to 80).

### Tobacco usage

Only 14(10%) of women reported tobacco use, 6(4%) reported smoking, 2(2%) reported use of a pipe and 6(4%) reported chewing tobacco. The average frequency of use of tobacco per day was 2.3 times ranging from one to four times a week.

### Age at menarche

The average age of menarche was 14 years (range 9 to 20). The mean ages at menarche among cases and controls were 14 (range 10 to 20) and 14 years (range 10 to 19), respectively.

The overall average age at menopause was 48 years (38 to 66). Among cases and controls, the average was 48 (40 to 66) and 49(38 to 55) years respectively.

### Contraceptive use

The number of participants that reported using contraceptives was 52 (36%) with an average duration of contraceptive use of 4 years (range 1 to 15). Of these, 26 (37%) of cases reported the use of oral contraceptives compared to 26 (36%) of controls.

### Red blood cell folate levels

**Figure 1:**
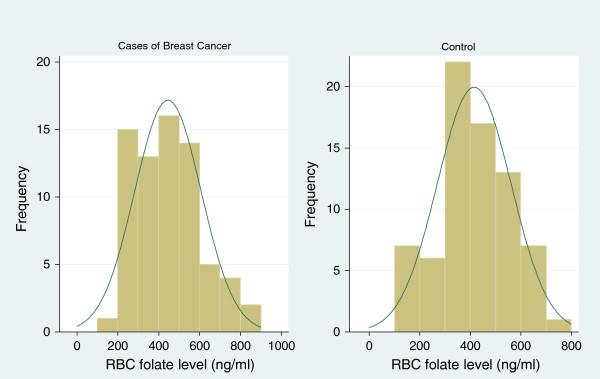
**Histogram of RBC folate levels among study participants.** RBC, red blood cells.

The mean RBC folate level was 430 ng/ml; range (105 ng/ml to 857 ng/ml) as shown in Figure 1. Low folate RBC was found in 90 participants (63%) (below 471 ng/ml – as was defined by this study) and 53 participants (37%) had normal RBC folate. The mean RBC folate for both cases and controls was below the cutoff of what was defined as normal. Cases had a mean RBC folate level of 445 (126 to 857 ng/ml) compared to 415 ng/ml, (105 to 734 ng/ml) among the controls.

### Vitamin B12 levels

**Figure 2:**
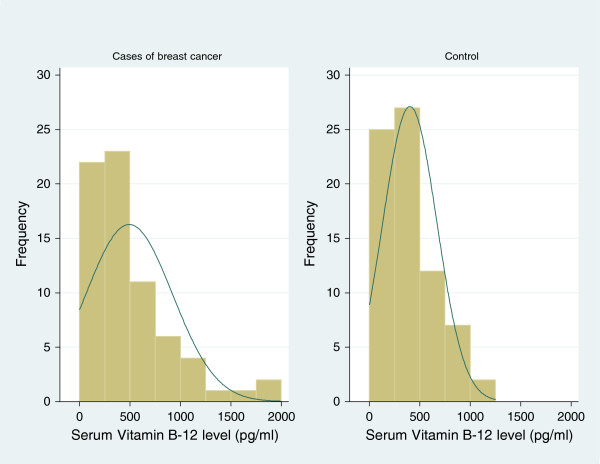
**Histogram of vitamin B12 folate levels among study participants.**

The average vitamin B12 level among women was 445 pg/ml (50 to 2000) with cases showing an average of 492 ng/dl (50 to 2000) and controls an average of 401 ng/ml (58 to 1114). Figure 2 shows the distribution of vitamin B12 levels among participants.

### Hemoglobin levels

**Figure 3:**
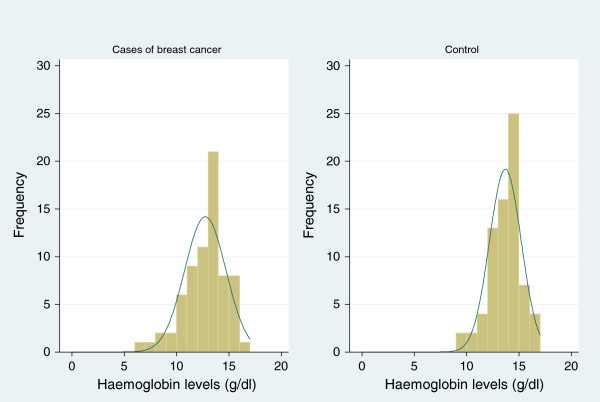
**Histogram of haemoglobin levels among study participants.**

The mean hemoglobin level was 13 g/dl (6.6 g/dl to 16.7 g/dl), with a mean of 12.7 g/dl (6.6 to 16.3) and 13.7 g/dl (9.2 to 16.7 g/dl) among cases and control respectively. See Figure 3 showing the distribution of hemoglobin among participants.

### Parity

Among cases, 6 participants were nulliparous (8%), 23 participants were of low parity (32%) and 43 were multiparous (60%). Among controls, 1 participant was nulliparous (1%), 19 participants had low parity (26%) and 53 were multiparous (73%).

### Body mass index

Among the cases, one participant was underweight (1%), 38 participants had normal weight (53%), 18 participants were overweight (25%) and 15 participants were obese (21%). Among controls, 4 participants were underweight 6%, 29 participants had normal weight (40%), 25 participants were overweight (34%) and 15 participants were obese (21%).

### Alcohol consumption

Among the cases, 43 (60%) reported alcohol use compared to 29 (40%) who reported no alcohol usage. Among the controls, 30 (41%) reported alcohol usage compared to 43 (59%) who reported no alcohol usage.

### Residence

Among the cases, 41 (59%) were from rural areas and 29 (41%) were from urban areas. Among the controls, 38 (52%) were from rural areas compared to 35 (49%) who were from urban areas.

### Study variables and their association with RBC folate levels

Contraceptive use and area of residence were the only characteristics associated with RBC folate levels (Table 2). Thirty five women (45%) from rural residences had normal folate levels compared to 18 (28%) women from urban residences. Thirteen (25%) women using contraceptives had normal folate levels compared to 40 (44%) women not using contraceptives.

**Table 2:** **The association between study participants and red blood cell folate levels**

Characteristics n = 145	Cases		Controls		***P***values*
	n = 72	%	n = 73	%	
**Age**					
20 – 39	22	24	8	15	
40 – 49	18	20	10	18	
50 - 59	25	28	19	36	
60 - 69	16	18	6	11	
70+	9	10	10	19	0.288
**Residence†**					
Rural	43	48	35	66.0	
Urban	46	52	18	34.0	0.040
**Body Mass Index**					
Underweight	3	03	2	4	
Normal	39	43	26	49	
Overweight	28	31	15	28	
Obese	20	22	10	19	0.913
**Contraceptive use**					
Yes	39	44	13	25	
None	50	56	40	76	0.021
**Menopause**					
Pre-menopause	38	42	21	40	
Post-menopause	52	58	32	60	0.661
**Parity**					
Null	5	6	2	4	
Low parity	27	30	15	28	
Multi parity	58	64	36	68	0.856
**Alcohol use**					
Yes	43	48	23	56.6	
No	47	52	30	43.4	0.308
**Taking tobacco**					
Yes	7	8	7	13.2	
No	82	92	46	86.8	0.320
**Hemoglobin**					
Severe anaemia	0	0	2	4	
Moderate anaemia	7	8	7	13	
Mild anaemia	8	9	5	9	
Normal	75	83	39	74	0.186
**Vitamin B12**					
Low	24	27	15	28	
Normal	66	73	38	72	0.832

*Chi square = p values.

Percentages may be = 100 due to rounding.

†Missing =2.

### Association between RBC Folate with breast cancer

There was no association seen between RBC folate levels and breast cancer among study subjects after controlling for confounding factors. Using logistic regression, the odds of having breast cancer among women with normal folate levels compared to those with low folate levels were 1.4 (95% CI 0.7 to 2.9), *P* value 0.291. Multivariate logistic regression showed that none of the potential confounding factors examined affected the association between folate levels and breast cancer.

### Association with other study variables

Examination of the crude association between other study variables using Pearson’s chi-squared test (χ^2^), showed that alcohol use was associated with having breast cancer(Table 3). Women who reported alcohol use had 2:1 odds of breast cancer compared to those with no alcohol use (95% CI 1.1 to 4.1 and *P* = 0.026). Details of the bivariate association of others factors with having breast cancer are shown in Table 3. Results of the association of RBC folate and vitamin B12 were also tabulated in this table.

**Table 3:** **Bivariate analysis of risk factors associated with having cancer of the breast at Mulago hospital**

Characteristics n = 145	Crude odds ratio (95%)	***P***values
**RBC folate level**		
Low	Referent	
Normal	1.4 (0.7– 2.9)	0.291
**Vitamin B12**		
Low	Referent	
Lower normal	0.9 (0.4 – 2.0)	0.835
Upper normal	1.3 (0.5 – 3.4)	0.599
**Age**		
20 – 39	Referent	
40 – 49	0.9 (0.3 – 2.4)	0.786
50 - 59	1.0 (0.4 - 0.5)	1.000
60 - 69	1.0 (0.3 – 3.0)	1.000
70+	1.1 (0.4 – 3.5)	0.858
**Residence**		
Rural	Referent	
Urban	0.8 (0.40 – 1.5)	0.434
**Body Mass Index**		
Underweight	Referent	
Normal weight	5.2 (0.6 – 49.4)	0.148
Overweight	2.9 (0.3 – 28.0)	0.362
Obese	4.0 (0.4 – 40.1)	0.239
**Contraceptive use**		
None	Referent	
Yes	1.0 (0.5– 2.1)	0.900
**Parity**		
Null	Referent	
Low parity	0.2 (0.1 – 2.0)	0.166
Multi parity	0.3 (0.1– 2.5)	0.253
**Alcohol use**		
No	Referent	
Yes	2.1 (1.1-4.1)	0.026
**Taking tobacco**		
No	Referent	
Yes	0.5 (0.2 – 0.6)	0.267

### Discussion

The aim of the study was to determine the association between RBC folate levels and breast cancer. There was no significant association found, the odds of having breast cancer among women with normal folate levels compared to those with low folate levels were 1.4 (95% CI 0.7 - 2.9) *P* = 0.291.

Folate plays a very important biochemical role in DNA synthesis, repair and methylation [3]. Folate is essential for regenerating methionine, the methyl donor for DNA methylation [7]. Folate is also essential for producing the purines and pyrimidine thymidylate required for DNA synthesis and repair [3]. Regeneration of methionine from homocysteine requires 5 methyl- THF as a substrate and Vitamin B12 as a cofactor [7]. Base modifications are involved in DNA packaging, with regions that have low or no gene expression usually containing high levels of methylation of cytosine bases [8].

Cytosine methylation produces 5-methylcytosine, which is important for chromosome inactivation. It has therefore been hypothesized that high intake of folate may reduce the risk of human cancers, including breast cancer [3].

Though not statistically significant, our findings suggest that females with low folate are less likely to have breast cancer when compared with females who have normal folate levels. This may mean that the observed higher RBC folate among cases may be associated with breast cancer. However, the observation of lower RBC folate among controls may also mean that low folate is protective against early breast carcinogenesis. These observations may lend themselves to other studies that have suggested that high folate is associated with breast cancer events, and that low folate is protective against breast cancer. This dual modulatory effect of folate on cancer has been observed by other epidemiologic, clinical, and animal studies [9]. The observation in this study is also in agreement with similar studies (both were case control studies and serum folate was used as a measure of association) by Wu *et al.*in 1999 and Rossi *et al.* in 2006 [10, 11]. The observation is also consistent with studies that did not find any association between dietary folate and breast cancer [9, 12, 13]. However, other studies suggest that high folate (serum folate, RBC folate and dietary folate) is associated with reduced risk of breast cancer [3, 7, 9].

### RBC folate levels among participants

We observed that females in our study had lower RBC folate than those from other countries, including China, Mexico and Australia where most of the studies on the association between RBC folate and breast cancer have been conducted [9, 12–15], though a recent Ugandan study suggested no difference [16]. This low folate is probably due to a low folate intake among the Ugandan population when compared to the western world.

Both the mean and range of RBC folate levels was slightly higher among cases, although this observation was not statistically significant. It is possible that the reason for this disparity lies in the fact that the breast cancer patients seen are prevalent rather than incident cases, and dietary changes due to nutritional improvement following illness may occur during the course of the disease which may affect RBC folate levels. On the other hand, since the difference among cases and controls is not statistically significant, the observed difference may not merit further scrutiny.

### Potential confounding factors

Confounding factors that were studied were not found to have an effect on the association between RBC folate and breast cancer. These included age, residence, alcohol use, BMI, tobacco use, hormonal contraceptive use, menopausal status, vitamin B12 levels, hemoglobin level, parity, and age at menarche and menopause.

### Association between BMI and area of residence

This study showed that 43% of study participants from rural areas were either overweight or obese compared to 62% overweight or obese participants from urban areas. This observation shows that living in urban settings is associated with either overweight or obese BMIs when compared to leaving in rural areas. Participants of a normal weight were found to differ considerably based on area, with 51% of participants coming from rural areas compared to only 34% coming from urban areas. Social and dietary habits may be the contributing factor, as participants from an urban area are more exposed to processed foodstuffs and drinks which may be rich in fats and calories, compared to participants from rural areas whose diet may mainly be based on cereals and fresh foods.

### Association between BMI and age

The average age of underweight participants was 64,that of normal weight participants was 52, that of overweight participants was 49, and that of obese participants was 52. Although underweight is observed to be associated with older age, other age categories were not associated with BMI. The observed low BMI among elderly participants may be due to the reduced general health status that is associated with this age group.

In this study, we used RBC folate levels instead of serum folate levels [11, 17] given that RBC folate levels are more representative of long-term folate status as compared to serum levels that assess short-term status.

### Limitations of the study

This study used the RBC folate cutoffs from the Australian population (471 ng/ml). Though this was found to be higher than the average RBC folate of the study controls, an earlier study observed that the status of folate levels among healthy young individuals was not different from that in Australia [16].

It was not possible to study only incident cases as patients are likely to seek care from other sources before they present at the national referral hospital, therefore the pre-morbid folate status may have been altered by interventions such as herbal medication (which may be rich in folate).

Dietary history was not obtained because of lack of a contextually appropriate tool. This would have provided a more accurate pre-morbid folate exposure.

Vitamin B6 (pyridoxine) was not studied yet it is an obligate cofactor, together with vitamin B12, for the conversion to the form of folate used for DNA synthesis and methylation.

Both the cases and controls may not have been true representatives of the general population given the fact that this was a hospital-based study, although this hospital is the only public institution with free comprehensive breast cancer services in the country and the majority of patients depend on free health care.

There could have been recall bias, for other variables such as age, contraceptive use though the main predictor variable was a biomarker which is not influenced by what a participant may recall.

## Conclusions

There was no association found between RBC folate levels and breast cancer.

## Abbreviations

RBC: red blood cells
DNA: deoxyribonucleic acid
WHO: World Health Organization
EDTA: ethylene diaminetetraacetric acid.
